# Enhancing biosensing sensitivity of metal nanostructures through site-selective binding

**DOI:** 10.1038/s41598-020-57791-4

**Published:** 2020-01-23

**Authors:** Na rae Jo, Yong-Beom Shin

**Affiliations:** 1 Department of Nanobiotechnology, KRIBB School, University of Science and Technology (UST), 34113 Daejeon, Republic of Korea; 2 BioNanotechnology Research Center, Korea Research Institute of Bioscience and Biotechnology (KRIBB), 34141 Daejeon, Republic of Korea; 3 BioNano Health Guard Research Center (H-GUARD), 34141 Daejeon, Republic of Korea

**Keywords:** Biosensors, Nanostructures

## Abstract

The localised surface plasmon resonance (LSPR) at the surface of metal nanostructures can induce a highly intense electromagnetic (EM) field, which is confined to the edges with big curvature or at narrow gaps between nanostructures. Therefore, the localisation of target molecules at these sites is crucial to achieve high sensitivity in LSPR-based biosensors. To this end, we fabricated a 40 nm high gold nano-truncated cone (GNTC) array using thermal nanoimprint lithography. As the EM field is most intense at the side surface and relatively weak at the top surface of GNTC, we improved the detection sensitivity by blocking the top surface with oxides to limit adsorption of antibodies and antigens to the top surface. We observed the difference in sensitivity by detecting α-fetoprotein (AFP) on the oxide-capped and uncapped GNTC arrays through sandwich immunoassay and enzymatic precipitation. The capped GNTC array exhibited higher detection sensitivity than the uncapped one. Particularly, six-fold enhancement of sensitivity was achieved in the serum sample. We used atomic force microscopy and electron microscopy to validate that the deposition of the oxides on the top surface of GNTC effectively blocked the adsorption of the biomolecules and the target molecules were preferentially adsorbed on the side surfaces.

## Introduction

Localised surface plasmon resonance (LSPR) is the collective oscillation of the conduction electrons, which is induced by the impingement of external photons on nano-sized metal structures^1,2^. The characteristics of LSPR, i.e. wavelength, intensity, and peak width of the resonance, depend on the compositional and morphological parameters, such as size and shape of the metal nanostructures^3,4^. Therefore, these parameters can serve as tuning parameters for the characteristics of LSPR. The variation in local environment of the nanostructures also changes the spectroscopic characteristics of LSPR, which implies that LSPR can be utilised as a monitoring tool for the molecular interaction in the vicinity of the nanostructures^5–9^.

Generally, the wavelength shift of LSPR is used as a quantitative measure of biosensing because it is proportional to the change in the dielectric constant due to the molecular adsorption on the surface of nanostructures^3,10^. The penetration depth of the electromagnetic (EM) field induced by surface plasmon (SP) is significantly small (<30 nm) in LSPR, and therefore, the EM field of LSPR is localised near the surface^11–14^. Thus, biodetection tools based on LSPR are considered suitable for biomolecular interactions that occur near the metal surface.

However, the EM field of plasmon is concentrated in particular regions of the metal nanostructures, and consequently, these regions exhibit the highest sensitivity to local changes in the refractive index (RI)^15–17^. Therefore, the detection sensitivity in LSPR-based biosensors can be improved by selectively immobilising the receptor molecules on the regions of high field density, so that the adsorption of the analyte is limited to these regions. This fact was addressed in a few recent studies^18–20^. In these studies, self-assembled chemistry was used to selectively immobilise the receptor molecules (such as antibodies) on both ends of a nanorod that responded to the LSPR of long wavelengths exhibiting high sensitivity. However, the number of target molecules that could be detected by the nanorod was limited because the tip of the rod was too narrow to immobilise two or more receptors. Furthermore, it is extremely difficult to achieve precise control of the distance between nanoparticles, as they are likely to aggregate during their surface modification and receptor immobilisation. Therefore, it may be impossible to discern a signal change due to the adsorption of target molecules on the surface of nanoparticles.

On the contrary, fabrication of nanostructures using top-down nanolithography has several advantages, such as size uniformity, easy adjustment of inter-structural spacing, and high reproducibility^21–24^. Unlike colloidal nanoparticles, these structures are free from undesired signal interference caused by plasmon coupling due to aggregation. However, there are no reports on the utilisation of selective adsorption of molecules to enhance the detection sensitivity of LSPR-based biosensors realised with such nanostructures.

We have previously reported that the density of the EM field induced on a nanodot is much higher at the edge or sidewall than at the top surface^25^. However, owing to its relatively large surface area, antibodies were preferably immobilised on the top surface of the gold nanodot. Consequently, antigens were also bound on this surface. In this study, we propose a method to increase the signal change by inducing selective adsorption of the receptor and analyte molecules on the high-density regions of the EM field. To this end, we have used thermal nanoimprint lithography and physical vapour deposition to fabricate uniform gold nano-truncated cone (GNTC) structures with high fidelity on a glass wafer. The intensity of the EM field is relatively weak at the top surface of the GNTC, and therefore, this region is blocked with an oxide material to prevent the undesirable adsorption of target molecules, thereby improving LSPR detection sensitivity. The signal changes caused by molecular adsorption are amplified by an enzyme-precipitation reaction^25^ to counteract the low sensitivity resulting from the short penetration depth of the EM field, particularly while using a large receptor such as an antibody. The biosensing sensitivity is improved by selectively immobilising the antibody on a specific region of high EM field intensity. Finally, we visualise this effect using atomic force microscopy and electron microscopy.

## Results and Discussion

### Fabrication and characterisation of GNTC array

Nanoimprint lithography is a powerful and high-speed method for fabricating metal nanostructures on a substrate, similar to self-assembly methods such as colloidal lithography and nanosphere lithography. Further, the array of nanostructures realised through imprinting has high levels of reproducibility and fidelity similar to those obtained with electron beam lithography (EBL) or focused ion beam (FIB) lithography. Here, we have fabricated arrays of gold nanostructures on a glass wafer using thermal nanoimprinting, in which it is relatively easy to remove a residual layer. When the height of the gold nanodot was low (~20 nm), most of the antibodies were immobilised on the top surface with an extremely weak EM field. Therefore, in order to maximise the signal change due to the adsorption of the target molecules, it was essential to increase the area of the side surface by increasing the height of the structure so that a large number of receptors could be immobilised on the side surface, which exhibits a highly intense EM field. Therefore, we fabricated gold nanodot arrays with various thicknesses over 20 nm. As shown in the TEM image of Supplementary Fig. S2, the entrance of the nanowell of the imprinted resin, which served as a mask, becomes narrower as the thickness of the gold deposition increases. Therefore, a truncated cone-shaped nanodot was naturally formed instead of a cylinder.

**Figure 1:**
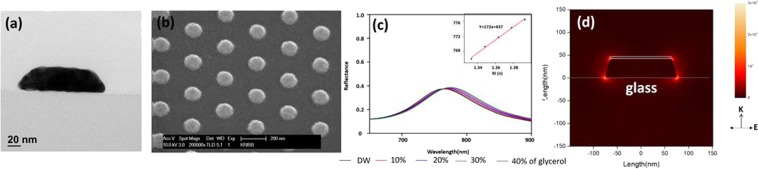
(**a**) Cross section of TEM image of capped GNTC. (**b**) Tilted SEM image of the capped GNTC chip. (**c**) The reflectance spectra of LSPR from the capped GNTC array at various concentrations of glycerol (0–40%). This plot shows the sensing characteristics of the capped GNTC. The inset depicts the linear relationship between the RI of the glycerol solution and LSPR wavelength (*λ*_cent_). (**d**) Intensity distribution of the electric field of capped GNTC on a glass substrate (RI = 1.52), calculated by finite-difference time-domain (FDTD). Here, the wavelength of light is 689 nm, and the surrounding medium is water (RI = 1.33). The wave vectors K and E represent the incident direction of the probe light and the polarisation direction, respectively.

The performance of an LSPR sensor, which monitors the change in RI near the surface of the metal nanostructure, is primarily decided by the effect of bulk RI sensitivity on the wavelength of plasmon resonance. Therefore, we examined the influence of height on the bulk RI sensitivity of GNTC arrays. We used 0–40% glycerol solution (with an RI value of 1.3329–1.3925) to measure the effect of change in RI on the resonance wavelength. It was observed that the bulk RI sensitivity decreased with the increase in height of the GNTC (Supplementary Fig. S3 and Table S1), which is in agreement with previous studies^26^. Therefore, while increasing the height of GNTC is desirable for immobilising maximum possible receptors on the side surface, it decreases the RI sensitivity. Accordingly, the height of GNTC was chosen as 40 nm, and a hexagonal array of GNTC with a bottom diameter (pitch) of 150 nm (300 nm) was fabricated. Further, we fabricated an SiO_2_-capped GNTC array by an additional deposition of 5 nm SiO_2_ to prevent the adsorption of receptor molecules on the top surface of the truncated cone. Figure 1 shows that the hexagonal array of GNTC was uniformly fabricated on a large area (Supplementary Fig. S4). The measured RI sensitivity of SiO_2_-capped GNTC was 172 nm RIU^−1^ (Fig. 1c), which was 7.5% less as compared to that of the uncapped one. However, this small reduction might not be a deteriorating factor for biosensing performance.

Usually, the electrons on the surface of a metal vibrate along the direction of the electric vector of the incident light. In the case of GNTC, the electron density is high at both ends of the metal structure, which is perpendicular to the direction of incident light. Particularly, the EM field is concentrated at the edge with a large curvature (Fig. 1d). The observation was similar in the case of uncapped GNTC without an SiO_2_ layer on the top surface (Supplementary Fig. S5).

### Sensitivity enhancement of the biosensor via site-selective immobilisation of antibodies

We chose α-fetoprotein (AFP) hepatoma marker as a detection target to improve the biosensing sensitivity by minimising the quantity of the antibodies that were immobilised to the top surface of GNTC. As the penetration depth of the evanescent field in LSPR is one-tenth of that in the propagating SPR, the sensitivity becomes severely limited when a target is detected by immobilising large receptors such as antibodies on the surface. Therefore, we applied sandwich immunoreaction and successive enzyme-precipitation reaction on the GNTC surface to increase the signal change, similar to that in earlier studies^25,27^. We also used the self-controlled scheme in which the target sample serves as a negative control (Supplementary Fig. S6), and defined the final variation in LSPR wavelength (Δλ) as the difference between the signal change (Δλ_s_) and the signal change (Δλ_c_) in the sample and control channels, respectively.

**Figure 2:**
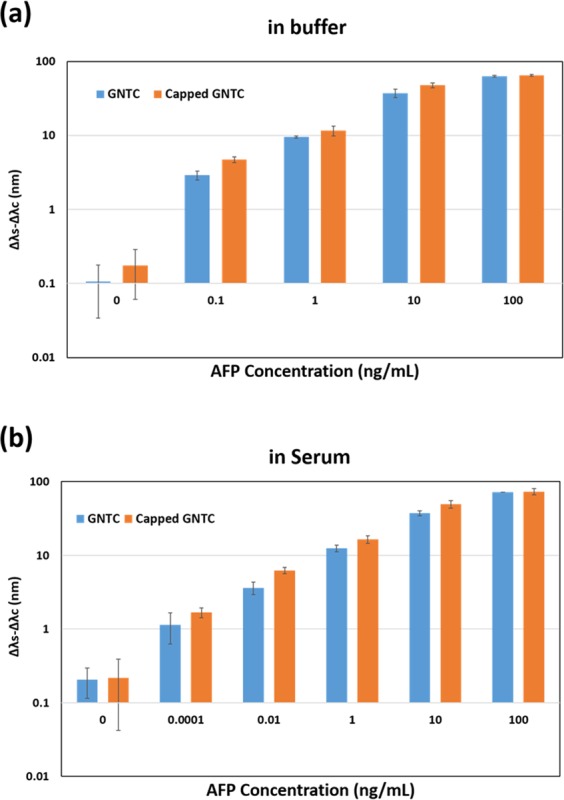
Comparison of the detection performance of the capped and uncapped GNTC for various concentrations of AFP in buffer and human serum. Δλ_s_ − Δλ_c_ is a final sensing value for each concentration. All experiments were repeated five times, and the error bars represent the standard deviations of the LSPR wavelength shifts for each concentration.

The effect of blocking the top surface of the nanostructure with oxides on the sensitivity was investigated by comparing the capped GNTC array (with blocked top surface) with the uncapped GNTC array (without blocked top surface). Figure 2a compares the signal change in LSPR for capped and uncapped GNTC arrays with the AFP concentration ranging between 100 pg ml^−1^ and 100 ng ml^−1^ in PBS buffer. The signal change was proportional to the AFP concentration in both cases. However, the signal changes were larger in the capped GNTC than in the uncapped one, and this disparity increased with the decrease in AFP concentration. This trend appeared more clearly in the serum sample with the lower concentration range (Fig. 2b). At a high concentration of 100 ng ml^−1^, the difference in Δλ of the two nanostructures was negligible. On the other hand, the gap between Δλ of the two nanostructures was ~33% at the AFP concentration of 10 ng ml^−1^, and it increased to ~62% at an even lower concentration of 0.01 ng ml^−1^. This can be explained as follows. At a high concentration of AFP, there were a large number of target molecules in the sample, and therefore, the molecules could fill both the top surface and the side surface and/or edges of the uncapped GNTC. As a result, the uncapped GNTC exhibited a similar wavelength change as the capped one. However, at a low concentration, there are not enough target molecules to bind with all the antibodies on the uncapped GNTC surface, and they are adsorbed primarily on the top surface. Therefore, a relatively small fraction of the target molecules are located at the side of the uncapped GNTC with a strong EM field, resulting in a smaller change in the resonance wavelength compared to that for the capped GNTC. From Fig. 2, the quantitative limit of detection (LOD) was calculated, using the following equation:

1 $${\rm{LOD}}=\frac{{S}_{c}^{m}+3\sigma -b}{a}$$

Here, *S*_c_^m^ and *σ* represent the average value of LSPR wavelength change $(\Delta {\lambda }^{0}:\Delta {\lambda }_{s}^{0}-\Delta {\lambda }_{c}^{0})$ in the buffer or serum sample (in the absence of AFP) and the standard deviation of Δ*λ*^0^, respectively. Variables *a* and *b* represent the slope and y-intercept of the linear fit for the Δ*λ* vs. AFP concentration plot (Fig. 2). In the PBS buffer sample, the LOD of the uncapped and capped GNTC arrays was 647 and 315 fg ml^−1^, respectively, thereby confirming that the sensitivity of capped GNTC was two times higher than that of uncapped GNTC. In the case of the serum sample, the LOD of the capped GNTC (~7 fg ml^−1^) was nearly six times better than the LOD of the uncapped GNTC (43 fg ml^−1^), implying that the sensitivity of the sandwich immunoassay could be improved by blocking the top surface of GNTC. Further, the detected AFP concentration was much lower in the serum than in the PBS buffer (Fig. 2). This may be attributed to the fact that AFP exists in the human blood naturally and the serum provided a similar physiologically active environment, where the antigen-antibody immune reaction could occur efficiently^28^.

### Confirmation of site-selective binding on GNTC chips

**Figure 3:**
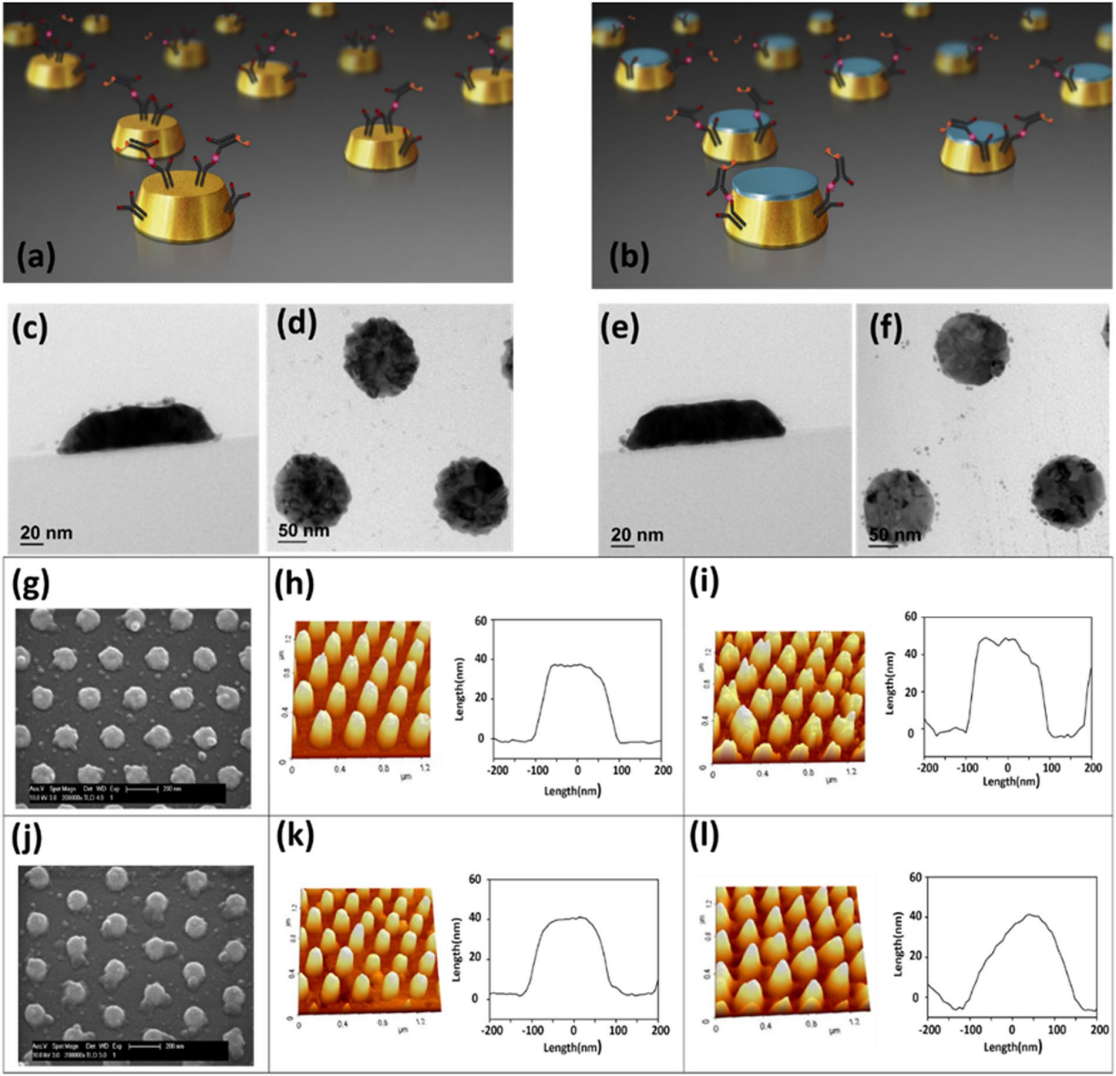
Schematic representation of binding of antibodies on (**a**) uncapped and (**b**) capped GNTC arrays. Frames (**c**,**d**) show the cross section and plane TEM image of uncapped GNTC arrays, respectively. The corresponding images for capped arrays are shown in frames (**e**,**f**). Frames (**g**,**j**) show SEM images of the uncapped and capped GNTC arrays after the final precipitation reaction, respectively. Frames (**h**,**k**) show the bare image and mean line profile data of the uncapped and capped GNTC arrays, respectively. These images were obtained by AFM. Frames (**i**,**l**) show the image and mean line profile data after precipitation reaction of the uncapped and capped GNTC arrays in 1 ng ml^−1^ AFP, respectively. These images were also obtained through AFM.

We have recently reported that the EM field was strongest at the edges of flat gold nanodots, which were fabricated on the substrate with nanoimprinting^25^. It was also confirmed that the antibodies were mainly immobilised on the relatively wider top surface and rarely on the side surfaces (which have a larger contribution to the signal change). Here, a capped GNTC array with blocked top surfaces was fabricated in a similar way as that discussed in the preceding section, and the sensitivity of AFP detection was enhanced using this array in an immuno-sandwich assay. Therefore, it is expected that the antibodies and antigens are preferentially located at the side surface for the capped GNTC (exhibits high EM field intensity) and at the top surface for the uncapped GNTC (exhibits low EM field intensity), as illustrated in Fig. 3a,b. However, this speculation needs to be verified with additional experiments. For confirmation, only the AFP antibody was fixed, and the roughness of the surface was confirmed through AFM (Supplementary Fig. S8). However, for the sake of clarity, we tried to visualise the binding of the antibody using Qdot. To this end, quantum dots conjugated with the anti-AFP antibody were reacted by adding them to the two types of bare array chips. The GNTC chips were then cut vertically and horizontally using an FIB, and TEM was then used to visualise the locations at which the antibodies were attached. For the uncapped GNTC chip, the quantum dots were located primarily on the top surface (Fig. 3c), and they were scarcely found at the side surface (Fig. 3d). However, for the capped GNTC, none of the quantum dots were found on the top, while several of them were surrounding the structures like a ring (Fig. 3e,f), which confirmed the constructional intent, i.e. site-selective binding on capped GNTC chips. Furthermore, to confirm that the precipitates, which are the final products of the reaction, were primarily accumulated on the sites containing antibodies and antigens, we visualised the morphological changes of GNTC using SEM and AFM. This was done for both types of GNTC arrays, which underwent the antigen-antibody reaction and the final enzymatic precipitation at the antigen concentration of 1 ng ml^−1^. As shown in the SEM image of Fig. 3, most of the precipitates accumulated on the top surface of the uncapped GNTC array (Fig. 3g). On the other hand, for capped GNTC arrays, the precipitates were stacked on the sides, and they appeared as if they were flowing and spreading out on the sides of the truncated cone (Fig. 3j). These aspects were more clearly verified in AFM experiments in which the changes in the average height and diameter were quantitatively analysed before and after the enzyme-precipitation.

The AFM measurement showed that the height (diameter) of the uncapped GNTC array increased by 10 nm (8 nm) after the enzyme precipitation (Fig. 3h,i). However, the average change in height of the capped GNTC array was 3 nm at most, which was a trivial increase as compared to the average change in diameter, which was 94 mm (from 224 nm to 318 nm) (Fig. 3k,l). Therefore, it was confirmed that most of the antibodies and antigens attached selectively on the side surface, and the precipitates from the ensuing enzyme reaction were localised there as well. Further, this maximised the effective change of RI in the GNTC arrays due to the adsorption of target molecules, thus improving the sensitivity of biosensing.

## Conclusions

Generally, the EM field originating from LSPR in metal nanostructures is unevenly distributed on the metal surface and localised on the edge with the largest surface curvature or at the narrow gap between metal surfaces facing each other. LSPR-based biosensors measure the minute changes in the RI near the metal surface. Therefore, it is crucial to fabricate the nanostructure for these sensors in such a way that most of the receptors and target molecules are confined to a region of high intensity EM field. In this study, we showed that the sensitivity of biosensing could be improved by fabricating a gold nanostructure using a top-down method, in which the low intensity regions were covered with a blocking material to facilitate the selective adsorption of the molecules in the high intensity regions. We fabricated the GNTC array on a 5-inch wafer with high fidelity, using thermal nanoimprint lithography. The height of the GNTC was selected as 40 nm, which was pertinent for the attachment of a sufficient number of antibodies while avoiding a significant decrease in the bulk RI sensitivity. We blocked the top surface of the GNTC with SiO_2_. Further, we fabricated the uncapped GNTC array with bare top surfaces and compared it with the capped one to examine the effect of the blocking on the biosensing performance of GNTC. Consequently, the LOD of the capped GNTC array for AFP detection was 315 fg ml^−1^ in the PBS buffer sample, which was nearly twice that of the uncapped one. In the case of the serum sample, the LODs of the capped and uncapped GNTC were 7 fg ml^−1^ and 43 fg ml^−1^, respectively, which implies the sensitivity in the former is six times higher as compared to that in the latter. The TEM analysis of GNTC after adsorption of the quantum dot-antibody conjugates demonstrated that the antibodies were selectively bound on the side surface of the capped GNTC. Furthermore, the AFM analysis showed that the precipitates, which were produced through the sandwich immunoreaction and successive enzyme reaction, accumulated preferentially at the sides of the capped GNTC and on the top surface of the uncapped GNTC. This proved that a site-selective attachment of the antibodies on the side surfaces could improve the biosensing sensitivity. This technique is potentially beneficial for diverse applications such as *in-vitro* diagnosis and environmental monitoring, which require extremely sensitive biosensing.

## Methods

The materials used, fabrication of the GNTC array, and optical characterisation of the GNTC array are described in detail in the Supplementary Information. The quantum dots used in this study had a narrow emission band with a maximum near 655 nm. The carboxyl group on the quantum dot surface was activated in a deionised water (DW) solution of 1-ethyl-3-(3-dimethylaminopropyl)-carbodiimide (EDC) (0.1 M) and N-hydroxysuccinimide (NHS) (0.025 M) for 13 min, and the activated quantum dots were reacted with 0.1 mg ml^−1^ anti-AFP in PBS for 2 h. To prevent nonspecific binding, bovine serum albumin (BSA) (100 mg ml^−1^ in 10 mM PBS) was added and reacted for 30 min. The solution was transferred to a clean centrifugal ultrafiltration unit (100 kDa cut-off). To remove any excess unbound protein, the solution was centrifuged at 1500 rpm for 30 min at least three times. The antibody-quantum dot conjugate solution was stored at 4 °C. The method for immobilisation of the antibody-quantum dot conjugate on the GNTC array was the same as that for the capture antibody (see Supplementary Information). The GNTC array was cut into thin slices to obtain vertical and horizontal cross sections using a dual beam FIB system (Helios NanoLab™), which enabled inspection by TEM.

## Supplementary information

Supplementary Information.

## Data Availability

All data generated or analysed during this study are included in this published article (and its Supplementary Information files).
