# Supplementary material for: Enhancing biosensing sensitivity of metal nanostructures through site-selective binding
Source: Sci Rep. 2020 Jan 23;10:1024. doi: 10.1038/s41598-020-57791-4 (PMC6978459; doi:10.1038/s41598-020-57791-4)
Supplement: Supplementary file 1 — Supplementary Information. [file 41598_2020_57791_MOESM1_ESM.docx]

SUPPLEMENTARY INFORMATION

Enhancing biosensing sensitivity of metal nanostructures through site-selective binding

*Na rae Jo^1,2^, Yong-Beom Shin^1,2,3^**

^1^Department of Nanobiotechnology, KRIBB School, University of Science and Technology (UST), 34113, Republic Korea

^2^BioNanotechnology Research Center, Korea Research Institute of Bioscience and Biotechnology (KRIBB), 34141, Republic of Korea

^3^BioNano Health Guard Research Center (H-GUARD), 34141, Republic of Korea

*ybshin@kribb.re.kr

MATERIALS

UV-curable perfluoropolyether (PFPE), which was used for replication of the master and imprint resin, and mr-I PMMA 35k200 were purchased from Solvay Solexis and Micro Resist Technology, respectively. Recombinant α-fetoprotein (AFP), anti-AFP antibody, and biotinylated anti-AFP antibody were obtained from Meridian Life Science. AFP Affinity Stripped Human Serum was purchased from Cone Bioproducts. 11-Mercaptoundecanoic acid (MUA), 1-ethyl-3-(3-dimethylaminopropyl)-carbodiimide (EDC), N-hydroxysuccinimide (NHS), bovine serum albumin (BSA), nitro blue tetrazolium (NBT), 5-bromo-4-chloro-3-indolylphosphatep-toluidine (BCIP), and streptavidin-alkaline phosphatase (ST-AP) were purchased from Sigma. Carboxylated quantum dots (QDs 655; CdSe core and ZnS shell) were purchased from Invitrogen.

FABRICATION OF GOLD NANO-TRUNCATED CONE ARRAY

The gold nano-truncated cone (GNTC) array was manufactured through a sequential procedure including nanoimprint lithography, metal deposition, and lift-off methods. The nanohole-patterned silicon master, which was used as the master mould, was fabricated by deep-UV lithography (ASML, PAS5500/700D KRF Scanner, 248 nm) and reactive ion etching (RIE, TCP-9400DFM). The diameter, pitch, and depth of the hole pattern were 150 nm, 300 nm, and 150 nm, respectively. A self-assembled monolayer (SAM; trichloro (1H,1H,2H,2H-perfluorooctyl) silane, Sigma-Aldrich, 97%) was added to the surface of the silicon master to prevent adhesion. The master surface was coated with UV resin (PFPE) to create a replica mould and then covered with polycarbonate (PC) film. The PC film on the master was pressed with a hand roller so that the hole pattern could be filled with the resin. The replica mould was cured by irradiating the PC film with UV (365 nm) light of 1 kW cm^-2^ intensity for 210 s. The thermoplastic resin (PMMA) was spin-coated on a glass substrate for imprint at a temperature (pressure) of 130°C (5 bar) for 2 h. The replica mould was detached from the substrate after cooling to 90°C. To protect the top region of the imprinted resin from O_2_ plasma etching, a 10 nm layer of chromium was deposited at a tilted angle of 70° during rotation in an e-beam evaporator. The residual layer was removed by O_2_ plasma etching (PINK Gmbh plasma-finish) at an O_2_ flow rate of 300 ml min^-1^ and power of 300 W for 6 min. Finally, a Ti/Au/SiO_2_ layer (1/40/5 nm) was deposited by e-beam evaporation, and lift-off was carried out by acetone solution for 5 min. The fabrication process is briefly described in Supplementary Fig. S1.


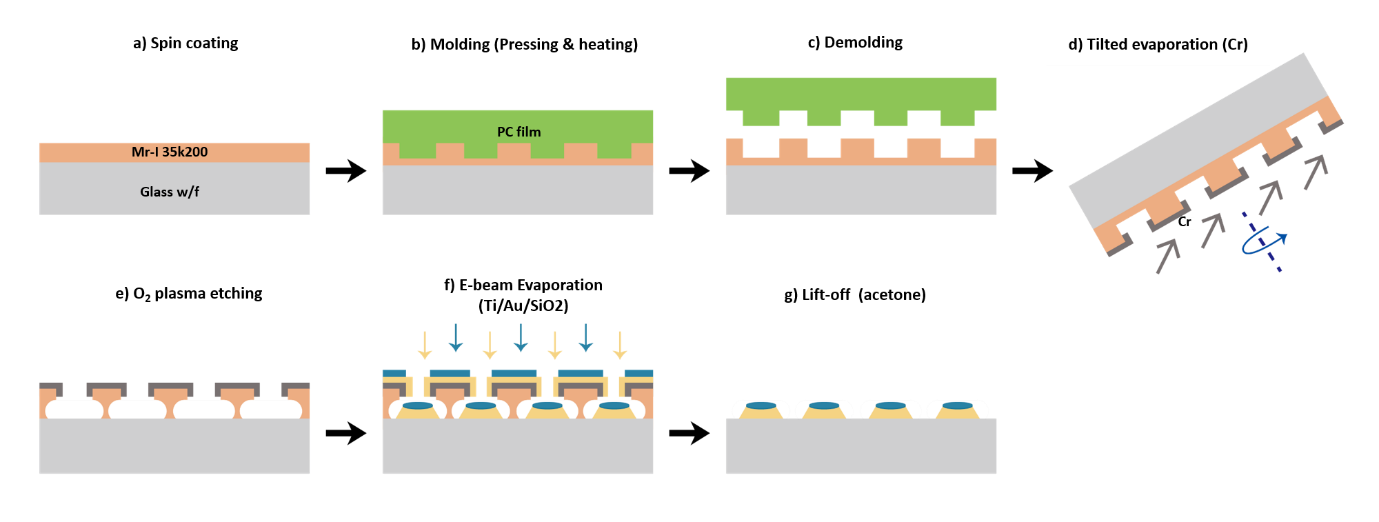


**Supplementary Figure S1.** Schematic illustration of the fabrication of the gold nano-truncated cone (GNTC) array on a glass substrate using thermal nanoimprint lithography (thermal-NIL).

PREPARATION OF THE GNTC SURFACE FOR THE DETECTION OF AFP

The GNTC array chips were cleaned with piranha solution (H_2_SO_4_/H_2_O_2_ = 3:1) at 90°C for 90 s to remove the surface contaminants. They were then rinsed with deionised water (DW) and dried with nitrogen gas. These chips were immersed in 10 mM MUA solution for 12 h. The carboxyl group on the MUA surface was activated in a DW solution of 0.1 M of EDC and 0.025 M of NHS for 13 min. Then, 0.025 mg ml^-1^ anti-AFP in PBS was injected, following which the chip was incubated overnight at 4℃. To prevent non-specific binding, PBS solution including 10 mg ml^-1^ of BSA was added and reacted for 30 min. The antigen-antibody reaction was achieved in PBS buffer and human serum containing recombinant AFP of different concentrations (100 fg ml^-1^-100 ng ml^-1^ and 10 pg ml^-1^-100 ng ml^-1^, respectively) for 30 min. The sandwich immunoassay was performed for 30 min with 1 μg ml^-1^ biotinylated anti-AFP and 1 μg ml^-1^ streptavidin-alkaline phosphatase in PBS buffer containing 1 mg ml^-1^ BSA. Finally, 1 M BCIP and 1 mg ml^-1^ NBT in AP buffer (100 mM Tris-HCl, 100 mM NaCl, 5 mM MgCl_2_) at pH 9.5 were added on the chip, and the enzyme-catalysed precipitation reaction was performed for 30 min. After each step, the samples were washed with PBS. The volume of all the samples was 50 μL.

OPTICAL CHARACTERISATION OF GNTC ARRAY

We applied back-reflection mode^1^ to measure the change in the wavelength of localised surface plasmon resonance (LSPR). Light traveling through an optical fibre was passed through the glass substrate to the GNTC chip and was reflected back from the structure. The scattered light was coupled back to the optical fibre along the same path.

Finite-difference time-domain (FDTD) simulations (Lumerical Inc., version 8.11) were used for optical characterization of the GNTC array. The geometric data of the GNTC were obtained from TEM and SEM images. Based on the information obtained from SEM and TEM, a micro-sized substrate was drawn first, followed by a gold nano truncated cone with a diameter of 150 nm at the bottom and 130 nm at the top. We used a planar EM wave as the incident light, which was sent from the negative z-direction and then reflected back from the GNTC, to simulate the actual experimental conditions. The wavelength of the light was in the range of 400-1000 nm, and periodic boundary conditions were applied to simulate the array structure. The reason for setting the boundary condition to periodic is that the GNTC chip is an array type, which repeats infinitely at the same interval. When the simulation setting for the structure is complete, the medium is set. The refractive index of the medium varies depending on whether it is in air, water, or other liquid state. Since the experiment was conducted in a PBS state, the refractive index (RI) designated water values. The RIs of the ambient medium and the substrate (glass) medium was 1.3329 (water) and 1.52, respectively. The RIs of gold and SiO_2_ were calculated by fitting the earlier reported values^2^ with Palik's theorem.^3^ The final step was to determine the mesh size that determines the accuracy of the simulation based on the size of the structure. The GNTC and its surrounding medium were divided into meshes of 0.5 nm size. After that, the simulation was run and the results analyzed.


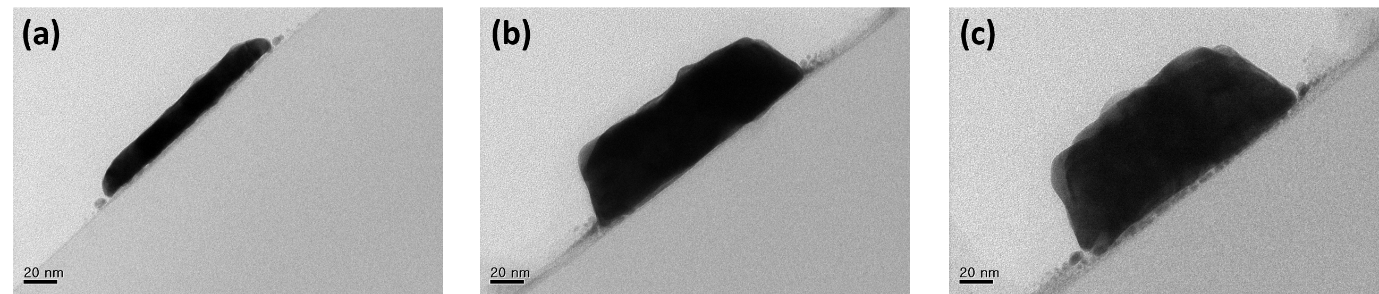


**Supplementary Figure S2.** TEM images (cross section) of uncapped GNTC. Gold heights in frames (a), (b), and (c) are 20 nm, 40 nm, and 60 nm, respectively.


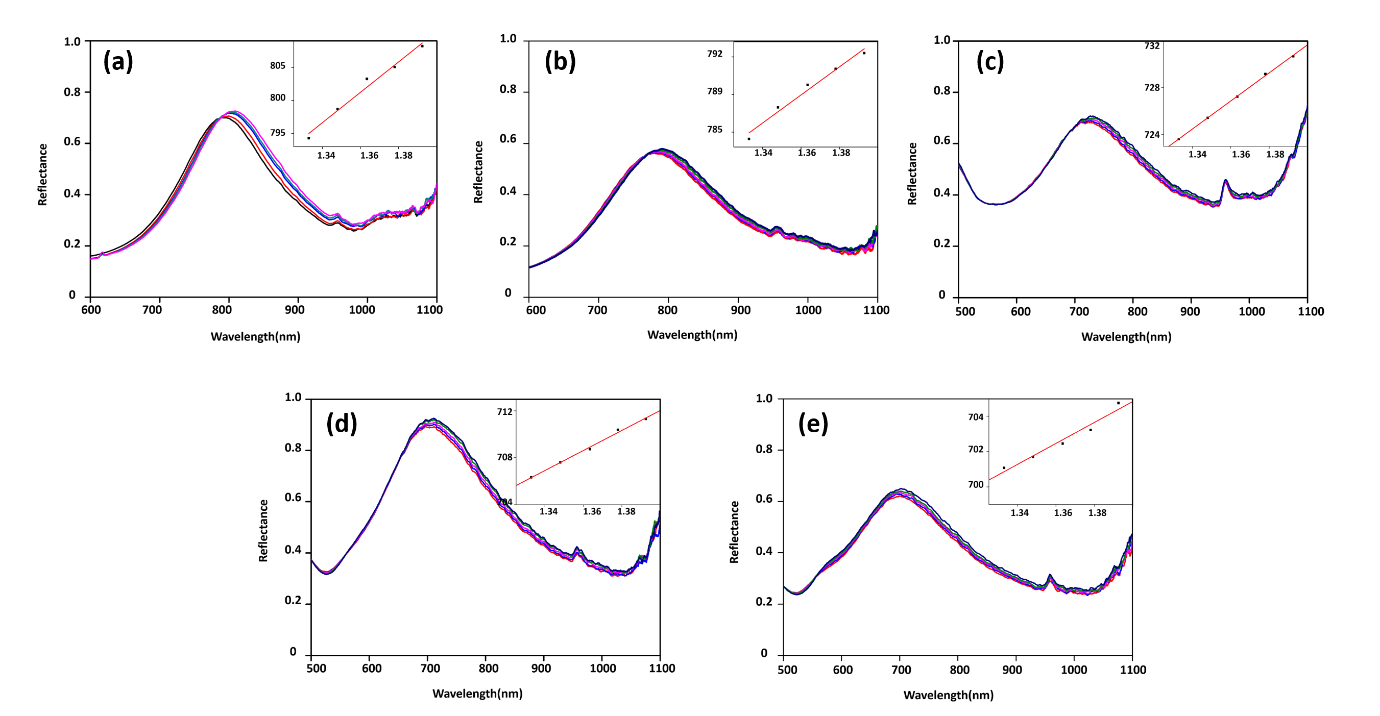


**Supplementary Figure S3.** Reflectance spectra obtained in various concentrations of glycerol solution (0-40%). Inset depicts the relation between RI sensitivity and LSPR wavelength (*λ*_cent_). Gold heights in frames (a), (b), (c), (d), and (e) are 20 nm, 40 nm, 60 nm, 80 nm, and 100 nm, respectively.

**Supplementary Table S1.** Bulk refractive index sensitivity as a function of gold height for GNTC chips.

| Au height | Sensitivity (nm/RIU) |
| --- | --- |
| 20 nm | 225 |
| 40 nm | 186 |
| 60 nm | 119 |
| 80 nm | 82 |
| 100 nm | 59 |


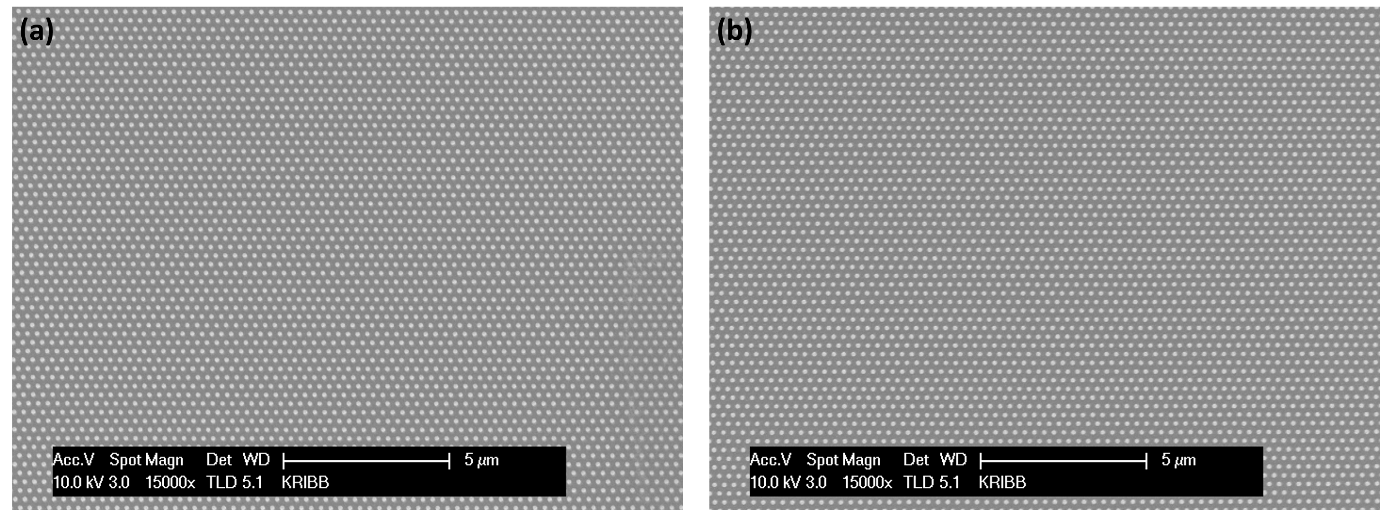


**Supplementary Figure S4.** SEM images of uncapped GNTC (a) and capped GNTC (b) (scale bar, 5 µm).

^
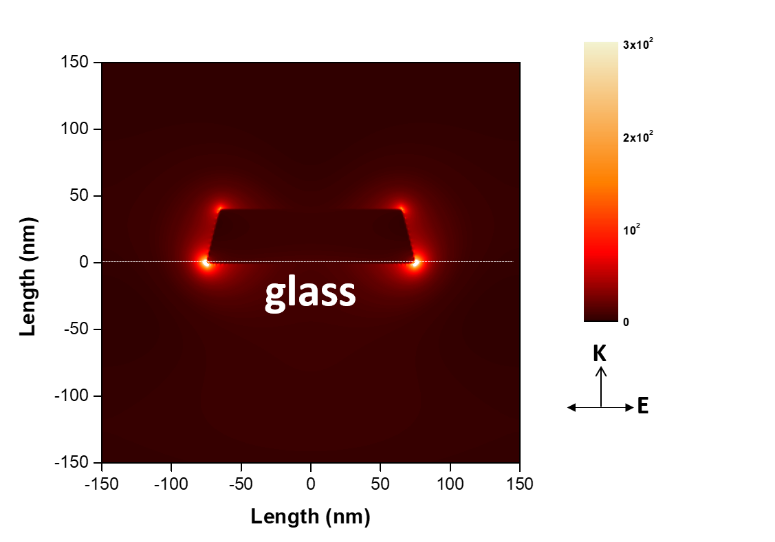
^

**Supplementary Figure S5.** Intensity (|E|^2^) distributions in uncapped GNTC array, obtained from FDTD calculations at a wavelength of 688 nm. The surrounding medium is water (RI=1.33). The wave vectors K and E represent the incident direction of the probe light and the polarisation direction, respectively.


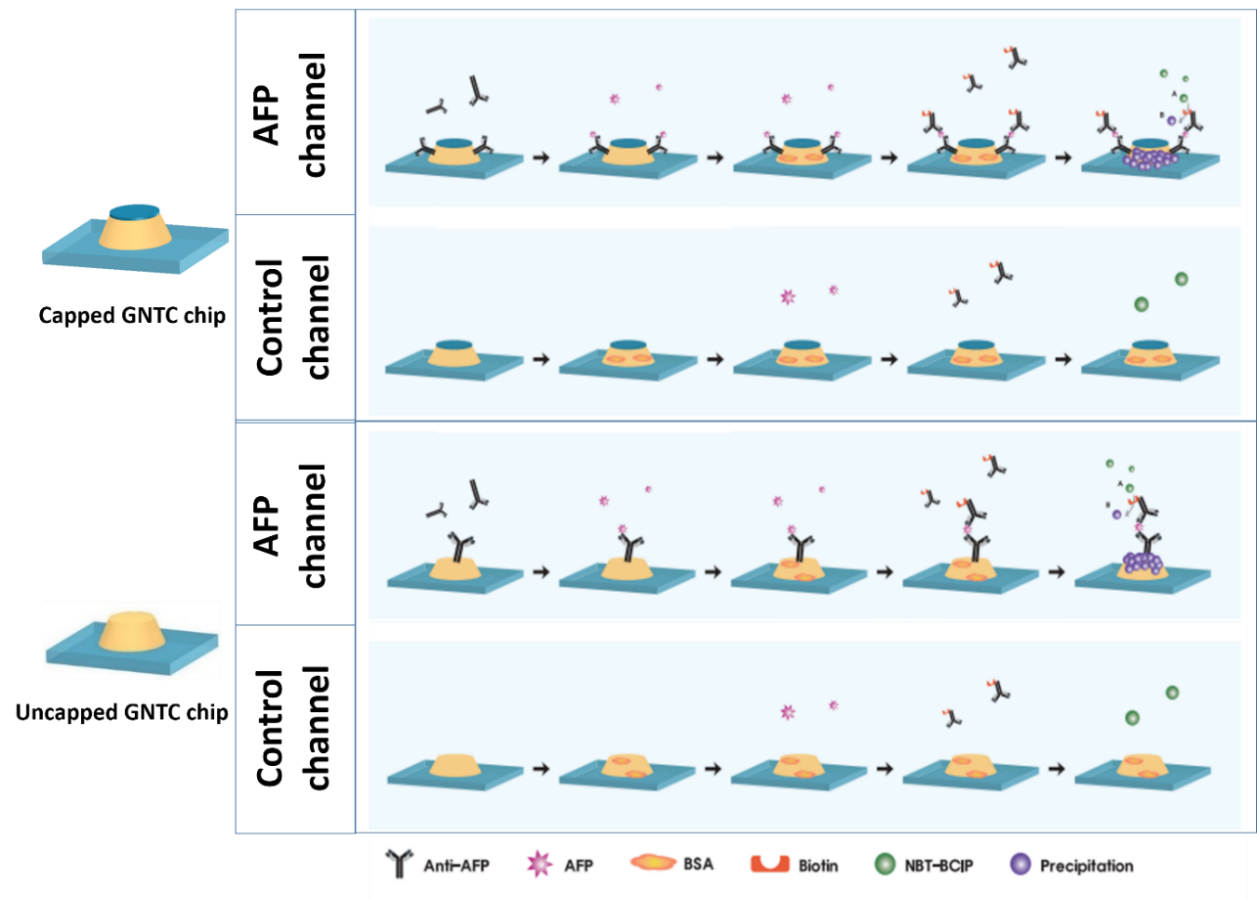


**Supplementary Figure S6.** Schematic illustration of the self-controlled detection system. The GNTC in the sample channel is immobilised with anti-AFP, while the control channel has no anti-AFP and is passivated with BSA. All other procedures are identical in all the channels.


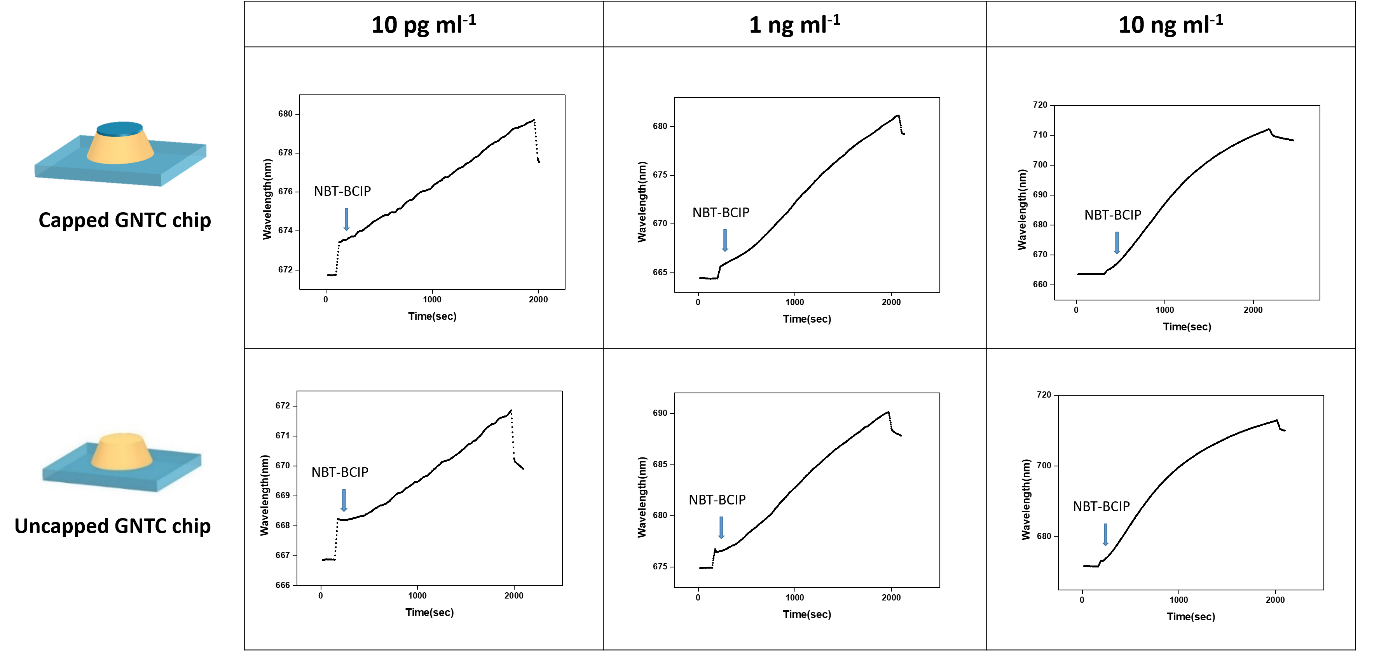


**Supplementary Figure S7.** Sensorgrams for the representative concentrations in serum experiments. These plots show the real-time graphs of enzyme-precipitation reactions. During the entire experiment, the antibody was immobilised on the chip surface, and the steps including antibody-antigen reaction and immobilisation of the enzyme were omitted.


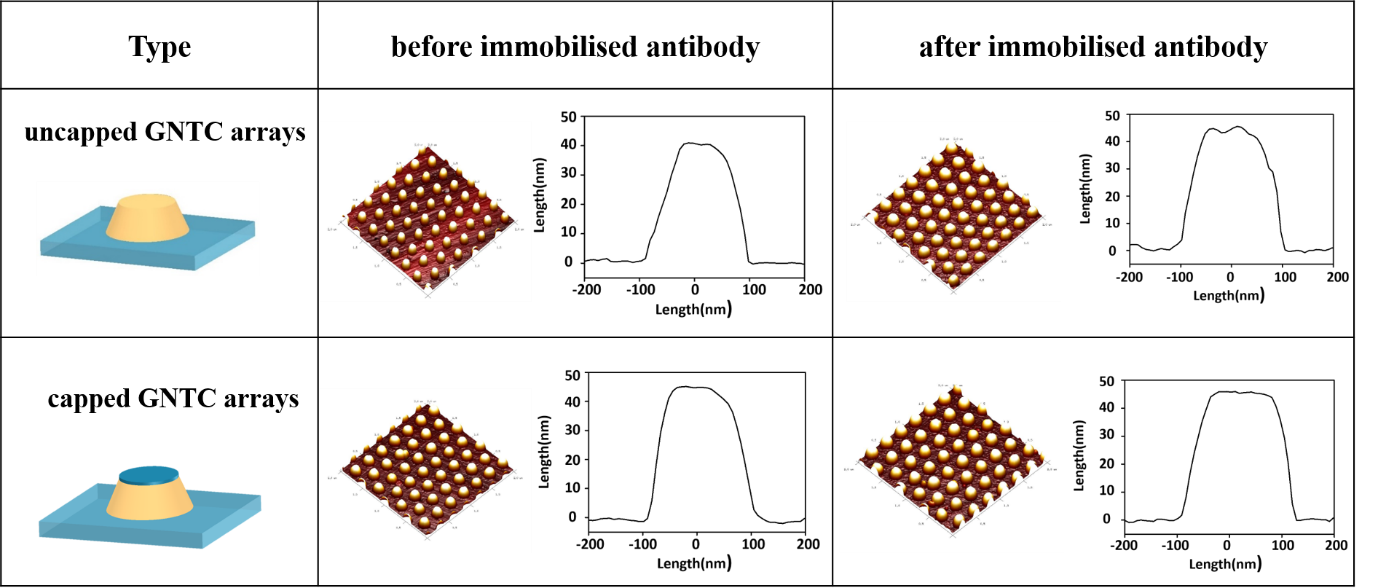


**Supplementary Figure S8.** AFM images. Frames (a) and (b) show the image and mean line profile data after reaction of the uncapped and capped GNTC arrays with 100 µg ml^-1^ AFP antibody, respectively.

**Supplementary Table S2.** The average height and diameter of the GNTC obtained from AFM measurements. These data indicate the change in height and diameter after immobilised the 100 µg ml^-1^ AFP antibody experiment.

|  | Avg. height (nm) | Avg. diameter (nm) |
| --- | --- | --- |
| GNTC before antibody immobilisation | 40.8 | 196.1 |
| GNTC after antibody immobilisation | 45.9 | 205 |
| Capped GNTC before antibody immobilisation | 45.7 | 195.7 |
| capped GNTC antibody immobilisation | 45.9 | 228.5 |

The AFM measurement showed that the height (diameter) of the uncapped GNTC array increased by 5nm after immobilized antibody. However, the capped GNTC was a increase as compared to the average change in diameter, which was 33 mm (from 195 nm to 228 nm). Therefore, it was confirmed that most of the antibodies attached selectively on the side surface.

**Supplementary Table S3.** Roughness of the top surface the GNTC obtained from AFM measurements. These data indicate the change in roughness after immobilisation of 100 µg ml^-1^ AFP antibody.

|  | Roughness (nm) |
| --- | --- |
| GNTC before antibody immobilisation | 40.59 ± 0.48 |
| GNTC after antibody immobilisation | 44.19 ± 1.99 |
| Capped GNTC before antibody immobilisation | 44.8 ± 0.86 |
| Capped GNTC antibody immobilisation | 45.62 ± 0.42 |

**Supplementary Table S4.** Averaged sample data corrected with the control data (Δλ_s_ – Δλ_c_) and the standard deviations of LSPR wavelength shifts for each concentration of AFP in PBS buffer. All experiments were repeated 5 times for each concentration.

| AFP concentration (ng ml^-1^) in PBS buffer (1% BSA) | GNCT chip | | Capped GNCT chip | |
| --- | --- | --- | --- | --- |
|  | △λ_s_ − △λ_c_ (nm) | Standard deviation | △λ_s_ − △λ_c_ (nm) | Standard deviation |
| Buffer | 0.104 | 0.065 | 0.102 | 0.057 |
| 0.1 | 3.102 | 0.269 | 4.711 | 0.435 |
| 1 | 9.523 | 0.254 | 11.547 | 1.892 |
| 10 | 37.125 | 4.833 | 47.511 | 3.972 |
| 100 | 62.891 | 1.508 | 64.599 | 2.013 |

**Supplementary Table S5.** Averaged sample data corrected with the control data (Δλ_s_ – Δλ_c_) and the standard deviations of LSPR wavelength shifts for each concentration of AFP in serum. All experiments were repeated 5 times for each concentration.

| AFP concentration (ng ml^-1^) in serum | GNCT chip | | Capped GNCT chip | |
| --- | --- | --- | --- | --- |
|  | △λ_s_ − △λ_c_ (nm) | Standard deviation | △λ_s_ − △λ_c_ (nm) | Standard deviation |
| Serum | 0.209 | 0.181 | 0.189 | 0.057 |
| 1.00E-04 | 1.143 | 0.511 | 1.765 | 0.323 |
| 0.01 | 3.621 | 0.681 | 5.859 | 0.613 |
| 1 | 12.457 | 1.269 | 15.706 | 1.954 |
| 10 | 37.215 | 2.899 | 49.541 | 6.252 |
| 100 | 72.155 | 0.286 | 73.253 | 6.976 |

**Supplementary Table S6.** The average height and diameter of the GNTC obtained from AFM measurements. These data indicate the change in height and diameter after precipitate reactions in the 1 ng ml^-1^ AFP experiment.

|  | Avg. height (nm) | Avg. diameter (nm) |
| --- | --- | --- |
| GNTC before antibody immobilisation | 38.7 | 218.7 |
| AFP-GNTC after enzyme precipitate reaction | 48.3 | 227.18 |
| Capped GNTC before antibody immobilisation | 40.5 | 223.9 |
| AFP-capped GNTC after enzyme precipitate reaction | 43.8 | 317.7 |

**Supplementary Table S7.** Comparison of different nano patterns.

|  | Nanopattern shape | Fabrication method | Measurement method | Target | LOD | Ref. |
| --- | --- | --- | --- | --- | --- | --- |
| 1 | Gold nano hole (d=440 nm) | E-beam lithography | Plasmonic | Vesicular stomatitis virus | 10^5^ PFU mL^-1^ | 4 |
|  | 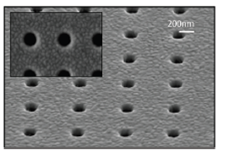 |  |  |  |  |  |
| 2 | Gold mushroom  (lattice constant=610 nm, pillar height=510 nm) | Photolithography | Plasmonic | Cytochrome c and AFP | 200pM and 15 ng ml^-1^ | 5 |
|  | 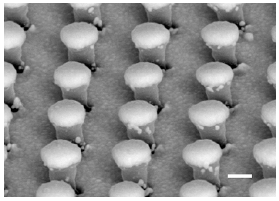 |  |  |  |  |  |
| 3 | Gold nano ring  (inner and outer diameter of 100 nm and 130 nm) | Nanosphere lithography | Plasmonic | DNA (25 nucleotides long) | 100nM | 6 |
|  | 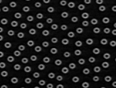 |  |  |  |  |  |
| 4 | Gold nanocup  (d=200 nm, 90 nm top and bottom Au layers and 80 nm TiO_2_ cavity layer) | Nanoreplica moulding process | Plasmon−cavity coupling | Carcinoembryonic antigen | 10 ng mL^-1^ | 7 |
|  | 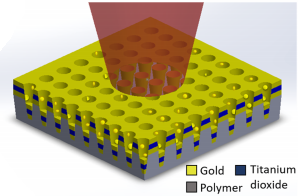 |  |  |  |  |  |
| 5 | Nanowell array (NWA)  (400 nm diameter) | Stepper | Electrochemical | Alkaline phosphatase | 0.13×10^−4^ U L^-1^ (μmol L^-1^) | 8 |
|  | 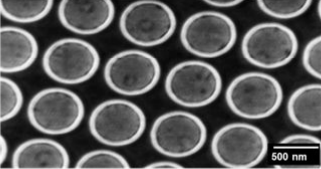 |  |  |  |  |  |

REFERENCES

1. Jo, N. R., Lee, K. J. & Shin, Y. B. Enzyme-coupled nanoplasmonic biosensing of cancer markers in human serum. *Biosens. Bioelectron.* **81**, 324–333 (2016).
2. Johnson, P. B. & Christy, R. W. Optical constants of the noble metals *Phys. Rev. B* **6**, 4370–4379 (1972).
3. Pierce, D. T. & Spicer, W. E. Electronic structure of amorphous Si from photoemission and optical studies *Phys. Rev. B* **5**, 3017–3029 (1972).
4. Yanik, A. A. *et al.* An optofluidic nanoplasmonic biosensor for direct detection of live viruses from biological media. *Nano Lett.* **10**, 4962–4969 (2010).
5. Shen, Y. *et al.* Plasmonic gold mushroom arrays with refractive index sensing figures of merit approaching the theoretical limit. *Nat. Commun.* **4**, 2381 (2013).
6. Huang, C., Ye, J., Wang, S., Stakenborg, T. & Lagae, L. Gold nanoring as a sensitive plasmonic biosensor for on-chip DNA detection. *Appl. Phys. Lett.* **100**, 173114 (2012).
7. Hackett, L. P. *et al.* Spectrometer-free plasmonic biosensing with metal–insulator–metal nanocup arrays. *ACS Sensors* **3**, 290–298 (2018).
8. Lee, J. *et al.* Measuring bone biomarker alkaline phosphatase with wafer-scale nanowell array electrodes. *ACS Sensors* **3**, 2709–2715 (2018).
